# A Metabolomic-Based Evaluation of the Role of Commensal Microbiota throughout the Gastrointestinal Tract in Mice

**DOI:** 10.3390/microorganisms6040101

**Published:** 2018-09-29

**Authors:** Yuri Yamamoto, Yumiko Nakanishi, Shinnosuke Murakami, Wanping Aw, Tomoya Tsukimi, Ryoko Nozu, Masami Ueno, Kyoji Hioki, Kenji Nakahigashi, Akiyoshi Hirayama, Masahiro Sugimoto, Tomoyoshi Soga, Mamoru Ito, Masaru Tomita, Shinji Fukuda

**Affiliations:** 1 Institute for Advanced Biosciences, Keio University, 246-2 Mizukami, Kakuganji, Tsuruoka, Yamagata 997-0052, Japan; yuppy@sfc.keio.ac.jp (Y.Y.); yumiko.sato.kj@riken.jp (Y.N.); mushin@sfc.keio.ac.jp (S.M.); wanping@sfc.keio.ac.jp (W.A.); tsukimi@sfc.keio.ac.jp (T.T.); kenji_nakahigashi@spiber.jp (K.N.); hirayama@ttck.keio.ac.jp (A.H.); msugi@sfc.keio.ac.jp (M.S.); soga@sfc.keio.ac.jp (T.S.); mt@sfc.keio.ac.jp (M.T.); 2 Graduate School of Media and Governance, Keio University, 5322 Endo, Fujisawa, Kanagawa 252-0882, Japan; 3 Central Institute for Experimental Animals, 3-25-12 Tonomachi, Kawasaki-ku, Kawasaki, Kanagawa 210-0821, Japan; rnozu@ciea.or.jp (R.N.); mueno@ciea.or.jp (M.U.); hioki@ciea.or.jp (K.H.); mito@ciea.or.jp (M.I.); 4 Health Promotion and Preemptive Medicine, Research and Development Center for Minimally Invasive Therapies, Tokyo Medical University, Shinjuku, Tokyo 160-8402, Japan; 5 Department of Environment and Information Studies, Keio University, 5322 Endo, Fujisawa, Kanagawa 252-0882, Japan; 6 Intestinal Microbiota Project, Kanagawa Institute of Industrial Science and Technology, 3-25-13 Tonomachi, Kawasaki-ku, Kawasaki, Kanagawa 210-0821, Japan; 7 Transborder Medical Research Center, University of Tsukuba, 1-1-1 Tennodai, Tsukuba, Ibaraki 305-8577, Japan; 8 PRESTO, Japan Science and Technology Agency, 4-1-8 Honcho Kawaguchi, Saitama 332-0012, Japan

**Keywords:** gastrointestinal tract, metabolome, metagenome, microbiota

## Abstract

Commensal microbiota colonize the surface of our bodies. The inside of the gastrointestinal tract is one such surface that provides a habitat for them. The gastrointestinal tract is a long organ system comprising of various parts, and each part possesses various functions. It has been reported that the composition of intestinal luminal metabolites between the small and large intestine are different; however, comprehensive metabolomic and commensal microbiota profiles specific to each part of the gastrointestinal lumen remain obscure. In this study, by using capillary electrophoresis time-of-flight mass spectrometry (CE-TOFMS)-based metabolome and 16S rRNA gene-based microbiome analyses of specific pathogen-free (SPF) and germ-free (GF) murine gastrointestinal luminal profiles, we observed the different roles of commensal microbiota in each part of the gastrointestinal tract involved in carbohydrate metabolism and nutrient production. We found that the concentrations of most amino acids in the SPF small intestine were higher than those in the GF small intestine. Furthermore, sugar alcohols such as mannitol and sorbitol accumulated only in the GF large intestine, but not in the SPF large intestine. On the other hand, pentoses, such as arabinose and xylose, gradually accumulated from the cecum to the colon only in SPF mice, but were undetected in GF mice. Correlation network analysis between the gastrointestinal microbes and metabolites showed that niacin metabolism might be correlated to Methylobacteriaceae. Collectively, commensal microbiota partially affects the gastrointestinal luminal metabolite composition based on their metabolic dynamics, in cooperation with host digestion and absorption.

## 1. Introduction

The intestinal microbiota consists of bacteria inhabiting the intestinal tract of mammals. They possess various functions, ranging from supporting the digestive function of their host to maintaining a robust immune system in the host [1]. Recently, there have been many reports about the functions of intestinal microbiota being related to host diseases such as obesity [2,3], diabetes [4,5,6], atherosclerosis [7,8,9], atopic dermatitis [10,11,12], immune system disorders [13,14], and brain function disorders [15,16,17,18,19]. Our gastrointestinal tract is a long organ system and its microbial diversity is dependent on each part and its corresponding functions. In the case of C57BL/6 mice fed with normal diet, a large percentage of Lactobacillaceae was observed in the stomach and small intestine, while the large intestinal microbiota is mainly composed of Bacteroidaceae, Prevotellaceae, Rikenellaceae, Lachnospiraceae, and Ruminococcaceae [20]. Some studies reported that small and large intestinal metabolome profiles were different, owing to the different composition of gastrointestinal microbiota [21,22]. However, metabolome profiling throughout the gastrointestinal tract, and its correlation with gastrointestinal microbiota, is not well understood. Therefore, we measured the gastrointestinal luminal metabolite concentrations across different parts of the intestinal tract in specific pathogen-free (SPF) and germ-free (GF) mice by using capillary electrophoresis time-of-flight mass spectrometry (CE-TOFMS) [23,24] and liquid chromatography-tandem mass spectrometry (LC-MS/MS). Furthermore, a 16S rRNA gene-based gastrointestinal microbiome analysis was conducted. 

## 2. Materials and Methods

### 2.1. Experimental Design

The animal experiments were performed using protocols approved by the Central Institute for Experimental Animals (CIEA) (Ethics numbers: 09043 (October 9. 2009) and 12036 (June 28. 2012)) (Kawasaki, Japan). Specific pathogen-free (SPF) male C57BL/6J mice were purchased from CLEA Japan Inc. Germ-free (GF) mice were raised at CIEA. During the duration of the experiment, the GF and SPF mice were each maintained in germ-free isolators at CIEA. GF and SPF mice were fed with a normal diet (CA-1, CLEA Japan Inc., Tokyo, Japan) *ad libitum* and housed individually in each cage. Diet and water were irradiated and autoclaved, respectively. Before dissection, these GF and SPF mice were acclimatized for 2 weeks in each germ-free isolator.

### 2.2. Sample Collection and Preparation for Metabolome Analysis

Twelve-week-old male GF and SPF mice (*n* = 3) were sacrificed, and their intestinal tracts were separated into 6 parts: Stomach (St), upper small intestine (Si1), lower small intestine (Si2), cecum (Ce), upper colon (Co1), and lower colon (Co2) (Supplementary Figure S1). The intestinal contents were stored at −80 °C until use. Extraction of intestinal metabolites was performed according to previously described methods, but with slight modifications [25]. The intestinal contents were run with 1 mL of 0.957 mM PBS containing 200 mM of internal standards (methionine sulfone and d-camphor-10-sulfonic acid) for metabolome analysis using a syringe. A total of 500 μL of the sample solution was mixed with 500 μL of 100% methanol, and then vortexed with 100 mg of 0.1 mm zirconia beads. The contents were centrifuged at 14,000 rpm for 5 min. The supernatants were transferred into centrifugal filter tubes (UltrafreeMC-PLHCC 250/pk for metabolome analysis, Human Metabolome Technologies, Tsuruoka, Japan) to remove the protein and lipid molecules, and then centrifuged at 4600× *g* for 3 h at 25 °C. The filtrates were centrifugally concentrated and dissolved in 30 μL of ultrapure water containing reference compounds (200 μM each of 3-aminopyrrolidine and trimesic acid) immediately before the CE-TOFMS analysis. Concentrations of sugars in the gastrointestinal luminal contents were determined by LC-MS/MS in the negative multiple reaction monitoring mode, using an API3000 triple quadrupole mass spectrometer (AB Sciex, Singapore). All the LC-MS/MS data were acquired using the Analyst Software (AB Sciex, Singapore).

### 2.3. LC-MS/MS Conditions

The mobile phase consisted of 0.5% (*v*/*v*) formic acid as solution A and acetonitrile as solution B. The gradient increased from 0% of solution B to 99%, taking 20 min, and then remained at 99% until 23 min. The flow rate used was 0.2 mL/min and the injection volume was 1 μL, as described previously [26].

### 2.4. CE-TOFMS Conditions

The CE-TOFMS conditions were the same as those described previously, but with slight modifications [27]. In the case of anionic metabolites, a commercially available COSMO(+) capillary (50 μm i.d. × 110 cm) (Nacalai Tesque, Kyoto, Japan), chemically coated with a cationic polymer, was used as the separation capillary. A total of 50 mM ammonium acetate solution (pH 8.5) was the electrolyte for the CE separation process. A new capillary was flushed successively with the running electrolyte, 50 mM acetic acid (pH 3.4), and then with the electrolyte again for 10 min. Before each injection, the capillary was equilibrated for 2 min by flushing with 50 mM acetic acid (pH 3.4) and then for 5 min by flushing with the running electrolyte. In the case of cationic metabolites, fused-silica capillaries (50 μm i.d. × 100 cm total length) filled with 1 M formic acid as the reference electrolyte was used as the separation capillary. After preparation, sample solutions (30 nL) were injected at 50 mbar for 3 s, and a voltage of 30 kV and −30 kV was applied. The capillary temperature was maintained at 20 °C, and the temperature of the sample tray was kept at below 5 °C. The sheath liquid, composed of methanol/water (50% *v*/*v*) and 0.1 μM hexakis (2,2-difluoroethoxy) phosphazene (Hexakis), was delivered at 10 μL/min. ESI-TOF-MS was conducted in the positive ion mode and 5 mM ammonium acetate (50% *v*/*v*)-methanol/water solution containing 0.1 μM Hexakis was delivered at 10 μL/min in the negative ion mode. The capillary voltage was set at 4 kV and 3500 V, for the cationic and anionic metabolites, respectively. In TOF-MS, the fragmenter, skimmer, and OCT RF voltage were set at 75, 50, and 125 V, respectively, in the positive ion mode, and at 100, 50, and 200 V, respectively, in the negative ion mode. A flow rate of drying nitrogen gas (heater temperature = 300 °C) was maintained at 10 L/min. In the case of the positive ion mode, automatic recalibration of each acquired spectrum was performed using reference masses of the reference standards ([^13^C isotopic ion of protonated methanol dimer (2MeOH + H)]^+^, *m*/*z* 66.06371) and ([protonated Hexakis (M + H)]^+^, *m*/*z* 622.02896). In the negative ion mode, automatic recalibration of each acquired spectrum was performed using reference masses of the reference standards ([^13^C isotopic ion of deprotonated acetic acid dimer (2CH3COOH–H)]^−^, *m*/*z* 120.03841), and ([Hexakis + deprotonated acetic acid (CH3COOH–H)]^−^, *m*/*z* 680.03554). Exact mass data were acquired at a rate of 1.5 spectra/s, over an m/z range of 50–1000. The alignment of the detected peaks was performed according to the *m*/*z* value and normalized migration time. 

### 2.5. Processing of Metabolome Data

Raw data were analyzed with our proprietary software: MasterHands [27] (Keio University, Fujisawa, Japan). Data was processed with noise-filtering, baseline correction, peak detection, and integration of the peak area from sliced electropherograms (*m*/*z* 0.02 width). Subsequently, the accurate *m*/*z* value for each peak detected within the time domain was calculated with Gaussian curve-fitting to the peak along the *m*/*z* axis. Spike noise, CE-specific noise showing small and narrow peaks, and low-quality (not peak-like shape) results were also removed. For the remaining features, metabolite identities were assigned by matching their *m*/*z* values and migration times with those of the standard compounds.

### 2.6. Metabolome Data Analysis

SIMCA-P 13.0.3. (Umetrics) and MultiExperiment Viewer (MeV TM4) [28] were used for orthogonal projections to latent structures-discriminant analysis (OPLS-DA) and heat map analysis based on K-means clustering of the data. Metabolic pathway analysis was performed according to Kyoto Encyclopedia of Genes and Genomes (KEGG) and significantly different (with *p*-values less than 0.1) metabolites between SPF and GF mice were analyzed by MetaCore Data-Mining and Pathway Analysis software (Thomson Reuters, New York, NY, USA) [29].

### 2.7. DNA Extraction, PCR Amplification, and Pyrosequencing of Gastrointestinal Luminal Contents

DNA extraction was performed as described previously, but with slight modifications [30]. Freeze-dried 10 mg gastrointestinal luminal contents were homogenized with mixture buffer (200 μL 10% SDS/TE, 200 μL of 3 M NaAc and 400 μL of phenol/chloroform/isoamyl alcohol) at 1500 rpm for 5 min and then centrifuged at 14,000 rpm for 5 min at 4 °C. After transferring the supernatant into new tubes, 400 μL of phenol chloroform/isoamyl alcohol was added and the mixtures were vortexed for 1 min. The samples were then centrifuged at 14,000 rpm for 10 min at 4 °C. After transferring the supernatant once again into new tubes, 800 μL of 100% EtOH was added and the samples were placed on ice for 15 min. The supernatants were centrifuged at 14,000 rpm for 5 min at 4 °C. The supernatants were then discarded and 500 μL of 70% EtOH was added to desiccate the pellet. The suspension was centrifuged at 14,000 rpm for 5 min at 4 °C. The supernatant was then discarded and the pellet was left to dry, after which 100 μL of TE was added and the samples were left at 4 °C overnight. The V1-V2 region (310 bp) of 16S rRNA genes were amplified from the genomic DNA samples using bacterial universal primer sets 27F (5′-AGAGTTTGATCMTGGCTCAG-3′) and 338R (5′-TGCTGCCTCCCGTAGGAGT-3′) [31,32]. PCR was performed with Tks Gflex DNA Polymerase (Takara Bio Inc., Kusatsu, Japan) according to the manufacturer’s specifications, and the amplification proceeded with an initial denaturation step at 98 °C for 1 min, followed by 20 cycles of 98 °C for 10 s, 55 °C for 15 s and 68 °C for 30 s, with a final extension step at 68 °C for 3 min [32]. They were visualized on agarose gels (2% in TAE Buffer) and purified using a QIAamp PCR Purification Kit. Purified PCR products were tagged with unique barcode labels using a forward primer (5′-CCATCTCATCCCTGCGTGTCTCCGACTCAGNNNNNNNNNNagagtttgatcmtggctcag-3′) containing the 454 primer A and reverse primer (5′-CCTATCCCCTGTGTGCCTTGGCAGT CTCAGtgctgcctcccgtaggagt-3′) containing the 454 primer B by PCR reaction under the following cycles: 2 min at 96 °C, 5 cycles of 96 °C for 30 s, 55 °C for 45 s, and 72 °C for 1 min, and a final extension of 72 °C for 10 min. Prior to sequencing, the 454-tagged PCR products were purified again with Agencourt AMPure XP (Beckman Coulter, Inc., Brea, CA, USA) and quantified using the Quant-iT PicoGreen DNA assay (Invitrogen, Carlsbad, CA, USA). Following quantization, the amplicons from each reaction mixture were pooled in equimolar ratios based on their concentrations and subjected to emulsion PCR to generate amplicon libraries. Amplicon pyrosequencing was performed on the Roche 454 GS Junior Titanium platform (Roche, Basel, Switzerland), according to the manufacturer’s specifications. The microbiome analysis data have been deposited at the DDBJ database (http://getentry.ddbj.nig.ac.jp/) under accession number DRA006964.

### 2.8. Analysis of Microbial 16S rRNA Gene Sequences

Following sequencing, the reads were de-multiplexed into samples according to the barcodes using the QIIME pipeline (v1.8.0) [33]. Barcode sequences and the last 20 bp were removed. Reads with low-quality scores (average *Q*-value <25), shorter than 200 bp, and longer than 1,000 bp were filtered out. The filter-passed reads were randomly selected from each sample and used for further analysis. These reads were clustered into operational taxonomic units (OTUs), which were selected on the Greengenes v. 13.8 database [34] with a 97% similarity threshold using UCLUST [35]. Beta-diversity was calculated using weighted and unweighted UniFrac on OTU tables, which were computed using the beta_diversity.py command in QIIME [36]. Principal coordinates were computed using principal_coordinates.py command in QIIME.

### 2.9. Statistical Analysis

Statistical analysis was performed with MATLAB version R2015a (The MathWorks, Inc., Natick, MA, USA). *p*-values < 0.05 were considered as statistically significant, with 0.05 < *p*-value < 0.1 as the tendency. The correlation network between metabolites and gut microbes based on Spearman’s correlation coefficient was constructed using graph.data.frame function of the igraph package in R statistical language, version 3.3.2. 

## 3. Results

### 3.1. Characteristics of Gastrointestinal Luminal Metabolites in SPF and GF Mice

**Figure 1:**
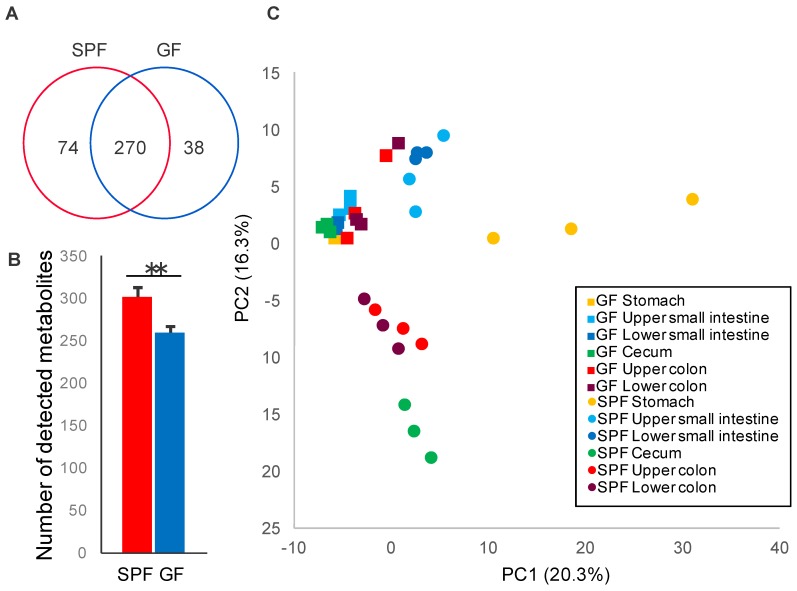
Gastrointestinal luminal metabolome profiles of specific pathogen-free (SPF) and germ-free (GF) mice. (**A**) Venn diagram showing the total number of gastrointestinal luminal metabolites detected in SPF and GF mice (*n* = 3). (**B**) Bar chart indicating the average number of detected gastrointestinal luminal metabolites in SPF and GF mice (*n* = 3). Error bars indicate standard error. *p*-values between SPF and GF mice were determined using Welch’s *t* test. **: *p* < 0.01. (**C**) Principal component analysis (PCA) score plots of the gastrointestinal luminal metabolome profiles in SPF and GF mice (*n* = 3). The PCA score plots were roughly separated by the presence of gut microbiota.

Metabolome analysis of gastrointestinal luminal contents by CE-TOFMS and LC-MS/MS identified a total of 382 metabolites from SPF and GF mice (Figure 1A). Our results indicated that the average number of detected metabolites in gastrointestinal contents of SPF mice were significantly higher than that of GF mice (Figure 1B). Additionally, 295, 269, 285, 300, 260, and 260 metabolites were detected in the stomach, upper small intestine, lower small intestine, cecum, upper colon, and lower colon, respectively, in SPF and GF mice (Supplementary Figure S2A). The average number of metabolites detected in the upper and lower colon were significantly higher in SPF mice than in GF mice (Supplementary Figure S2B), suggesting that colonic microbiota may produce unique type of metabolites only in SPF mice. To visualize the differences between the gastrointestinal luminal metabolome data of the GF and SPF mice, we performed a PCA. The PCA score plots of the metabolome data for the GF and SPF mice were roughly separated and clustered only in GF mice (Figure 1C), suggesting that the gastrointestinal luminal metabolome profiles of SPF mice had higher diversity than those of GF mice due to colonization of gastrointestinal microbiota, which may drive gastrointestinal luminal metabolome environment.

### 3.2. Comparison of Luminal Metabolome Profiles among Each Part of the Gastrointestinal Tracts in SPF and GF Mice

**Figure 2:**
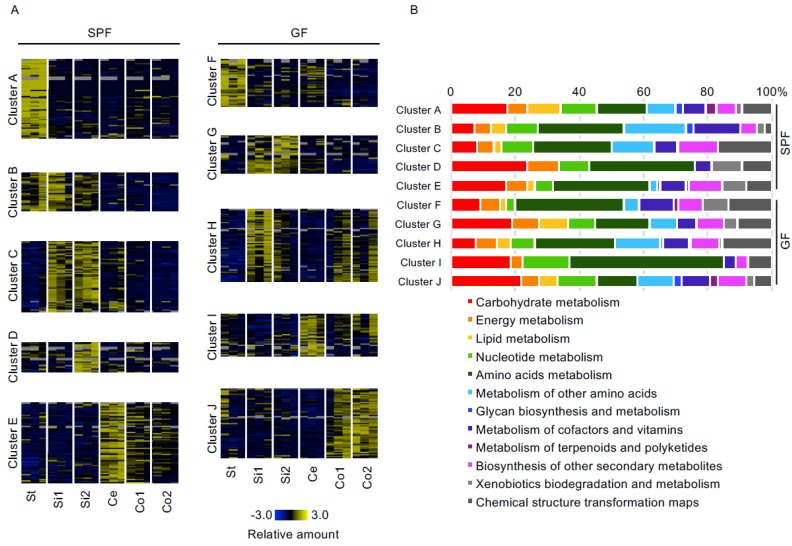
Differences in gastrointestinal luminal metabolites among different parts of the gastrointestinal tract of SPF and GF mice. (**A**) Heatmap of luminal metabolites categorized into five groups by using K-means analysis. Columns and rows correspond to gastrointestinal parts and metabolites detected by CE-TOFMS and LC-MS/MS, respectively. These profiles were normalized to a relative value between −3 and 3. The colors indicate the z-score values calculated for the SPF and GF mice separately (*n* = 3). St: Stomach, Si1: Upper small intestine, Si2: Lower small intestine, Ce: Cecum, Co1: Upper colon, Co2: Lower colon. (**B**) Bar graphs showing the composition of the KEGG categories based on the luminal metabolites in each cluster.

To focus on the metabolome features in each part of the gastrointestinal tract, we conducted K-means clustering based on the z-scores of the gastrointestinal luminal metabolome data (Figure 2A). In SPF mice, each cluster indicates the specific features of the gastrointestinal organ. Some metabolites at their highest concentrations in the stomach were clustered as cluster A, those in the stomach or upper and lower small intestine as cluster B, those in the upper and lower small intestine as cluster C, those in the lower small intestine as cluster D, and those in the cecum as cluster E. On the other hand, metabolites observed in GF mice were not separated clearly into clusters representing their respective specific intestinal parts, unlike in SPF mice. Metabolites observed at their highest concentrations at the stomach and small intestine were clustered as cluster F and cluster G, respectively, and those in the upper small intestine and colon, and in the cecum and colon were clustered as cluster H and cluster I, respectively, while those in the colon were clustered as cluster J. All metabolites in their respective clusters were categorized accordingly to metabolic pathways in the KEGG database (Figure 2B). We found that the profile of KEGG-based categorized clusters were not much different between SPF and GF mice.

In case of SPF mice, the results of the MetaCore analysis have suggested that the metabolites that were significantly more abundant than those in GF mice may be related to amino acid and nucleotide metabolism, and its regulation. In the stomach and small intestine, the metabolites that existed in high concentrations were related to amino acid metabolism and regulation, such as glycine, serine, cysteine, and threonine metabolism (betaine, choline, dimethylglycine, choline phosphate, creatine, 5-amino-levulinic acid), taurine and hypotaurine metabolism (taurine), beta-alanine metabolism (beta-alanine), and arginine metabolism (urea, guanidinoacetate, creatine, spermidine) (Supplementary Table S1). In the cecum and upper colon of SPF mice, the differentially abundant metabolites, compared to those in GF mice, were related to nucleotide metabolism and regulation, such as TTP metabolism (uracil, thymine, 2′-deoxythymidine), CTP/UTP metabolism (uridine, uracil, cytidine, beta-alanine, UMP, and CMP), and UMP biosynthesis. In the lower colon, the metabolites present were related to amino acid metabolism and regulation, such as histidine-glutamate-glutamine metabolism (urocanic acid, succinic acid), and tricarbonic acid cycle (succinic acid). In the case of GF mice, there were a few metabolites detected. In the stomach and upper small intestine, the highly abundant metabolites were related to nucleotide metabolism and regulation, such as GTP-XTP metabolism (guanosine, guanine) and dGTP metabolism (2’-deoxy-guanosine). In the lower small intestine, the metabolites detected in high concentrations were related to nicotine action, such as nicotine metabolism in the liver (S-nornicotine). In the cecum and colon, the metabolites were related to amino acid metabolism and regulation, such as proline metabolism (4-hydroxy-l-proline), arginine metabolism (guanidinoacetate), and glycine, serine, cysteine, and threonine metabolism (guanidinoacetic acid). Although there are overlaps in the metabolic pathways between the SPF and GF mice, the differences in specific metabolites exist in the gastrointestinal tracts of the SPF mice (Supplementary Table S1), indicating that gastrointestinal microbiota contributes to production of specific metabolites in the gastrointestinal tract. 

**Figure 3:**
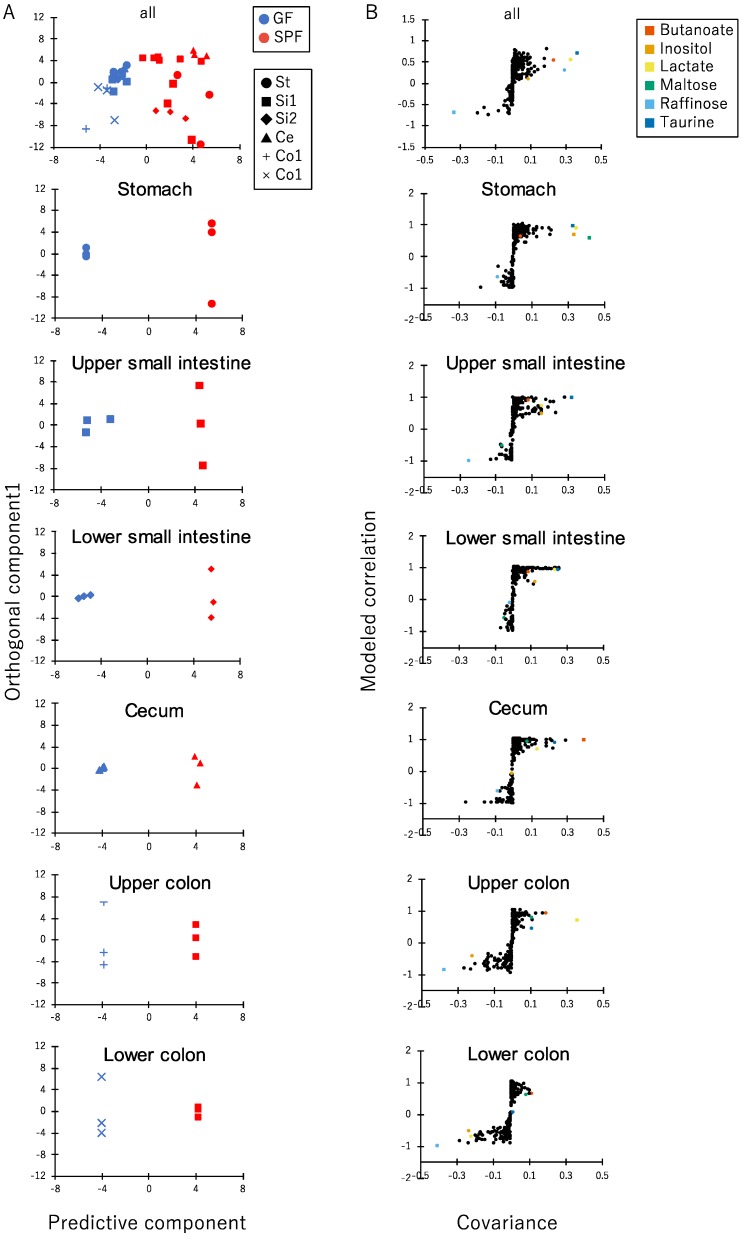
Orthogonal projections to latent structures-discriminant analysis (OPLS-DA) of the luminal metabolome profiles of the gastrointestinal tract of SPF and GF mice. (**A**) The result of OPLS-DA. The red and blue colors indicate the SPF and GF mice, respectively (*n* = 3). (**B**) S-plot for OPLS-DA of each part of the gastrointestinal tract. The criteria for the models were as follows: Cross-validated predicted ability Q2(Y) = 1 and total explained variance R2(X) = 1. The values of cross-validated predicted ability and total explained covariance for the stomach, upper small intestine, lower small intestine, cecum, upper colon, and lower colon in the constructed models were: Q2(Y) = 0.96 and R2(X) = 0.93, Q2(Y) = 0.99 and R2(X) = 0.98, Q2(Y) = 0.99 and R2(X) = 0.89, Q2(Y) = 1 and R2(X) = 1, Q2(Y) = 1 and R2(X) = 1, respectively.

In order to determine the signature of different metabolites between GF and SPF mice, we conducted OPLS-DA evaluation on each part of the luminal metabolome profiles of SPF and GF mice (Figure 3A). The S-plots showed that some metabolites contributed to the separation between SPF and GF mice (Figure 3B and Supplementary Table S2). The concentrations of taurine (81.9-fold in the stomach and 7.2-fold in the upper small intestine), lactate (7.7-fold in the stomach, 4.5-fold in the upper colon), inositol (11.1-fold in the stomach), maltose (811.0-fold in the stomach), and butanoate (1374.2-fold in the upper colon) were higher in the SPF mice than in the GF mice. On the other hand, the concentration of raffinose (3680.3-fold in the colon) was higher in the GF mice than in the SPF mice. Lactate and butanoate are well known microbial fermentation products and exert profound influence on host physiology [37,38]. Taurine is a component of primary conjugated bile acids such as taurocholic acid. It has been reported that gastrointestinal microbiota metabolizes taurocholic acid to cholic acid and releases taurine, and this bile acid metabolization largely contributes to development of obesity through FXR signaling [39,40]. Maltose is a disaccharide which is generally produced from starch and absorbed by host small intestinal epithelial cells. Raffinose is a trisaccharide which generally metabolized by gastrointestinal microbiota. Based on these findings, the signature of different metabolites between GF and SPF mice is related to the presence of gastrointestinal microbiota. 

### 3.3. Correlation between Gastrointestinal Microbes and Metabolites in SPF Mice

**Figure 4:**
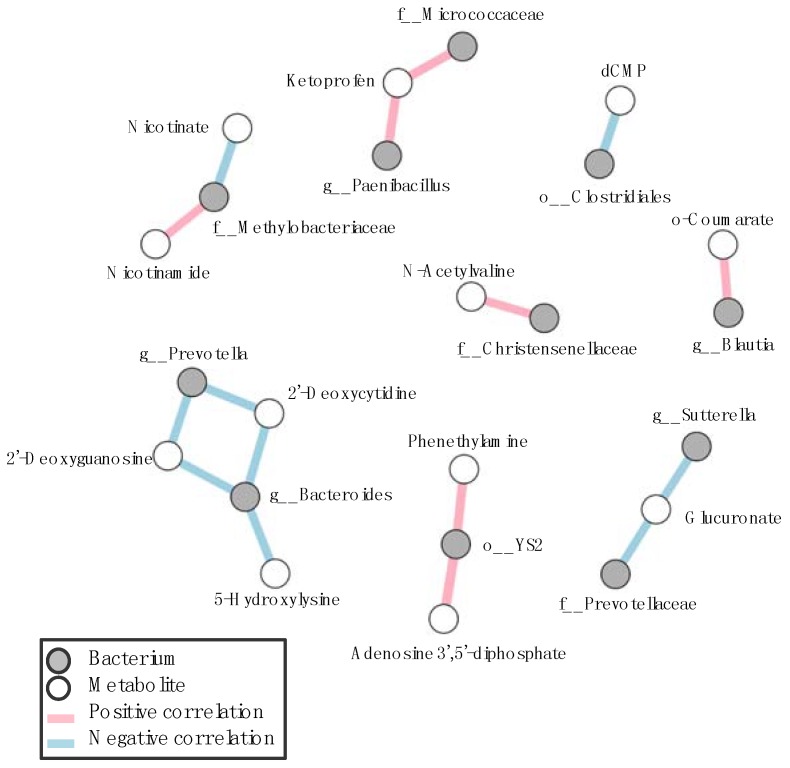
Correlation networks between gastrointestinal microbes and metabolites in SPF mice. Correlation plots of pairwise correlations among gastrointestinal microbe-metabolite were calculated by Spearman’s rank correlation coefficient. Positive and negative correlations at the cut off level of the *p* value of 0.05 are shown as red and blue lines, respectively. Gray and white circles indicate gastrointestinal microbes and metabolites, respectively.

We next conducted a microbiome analysis of the gastrointestinal tract of SPF mice and observed distinct microbiome profiles amongst different parts of the gastrointestinal tract (Supplementary Figure S3A,B). Consistent with the previous reports, the relative abundance of Lactobacillaceae was higher in the upper gastrointestinal tract [20]. Correlation network analysis between gastrointestinal microbes and metabolites revealed that several microbes and metabolites were significantly correlated (Figure 4). Nicotinate–nicotinamide metabolism, known as vitamin B3 metabolism, is significantly correlated with Methylobacteriaceae. It has been reported that α proteobacteria has a nicotinate–nicotinamide metabolic enzyme [41], suggesting that Methylobacteriaceae is considered to be one of the nicotinate–nicotinamide metabolizing microbes in the gastrointestinal tract of SPF mice. Several amino acid derivatives, such as 5-hydroxylysine and *N*-acetylvaline, were also significantly correlated with *Bacteroides* and Christensenellaceae, respectively.

### 3.4. Gastrointestinal Luminal Features of Sugars and Amino Acids in SPF and GF Mice

**Figure 5:**
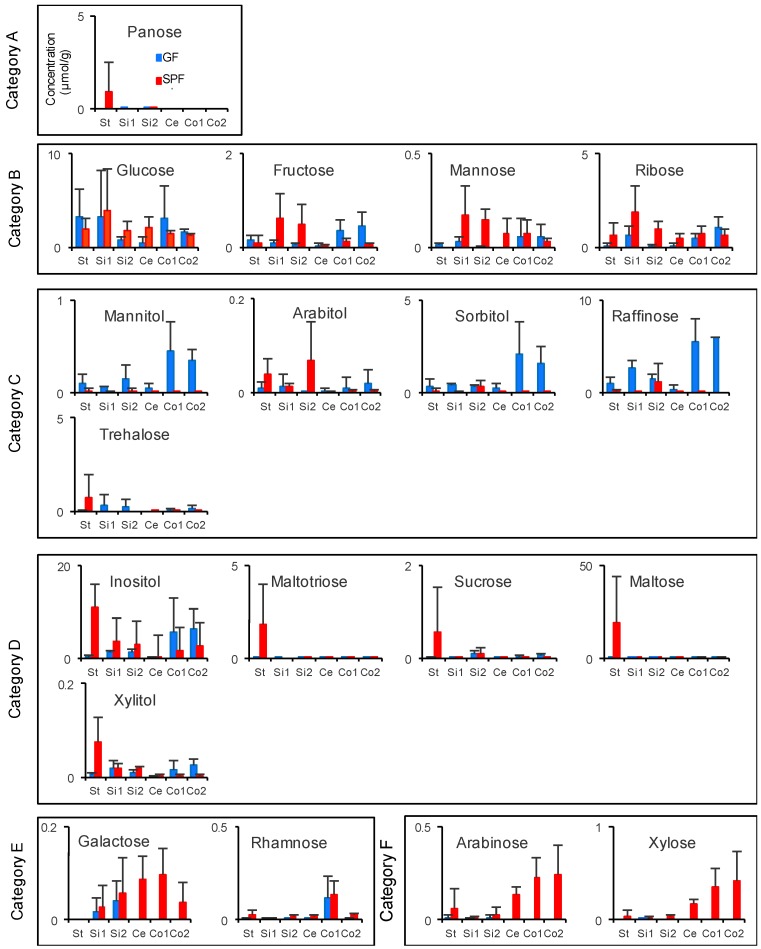
Luminal carbohydrate profiles of the gastrointestinal tract of SPF and GF mice. Line graphs show the average concentrations of carbohydrates in the gastrointestinal contents. The red and blue lines indicate SPF and GF mice, respectively. The error bars represent the standard error (*n* = 3). St: Stomach, Si1: Upper small intestine, Si2: Lower small intestine, Ce: Cecum, Co1: Upper colon, Co2: Lower colon. Unit: μmol/g.

**Figure 6:**
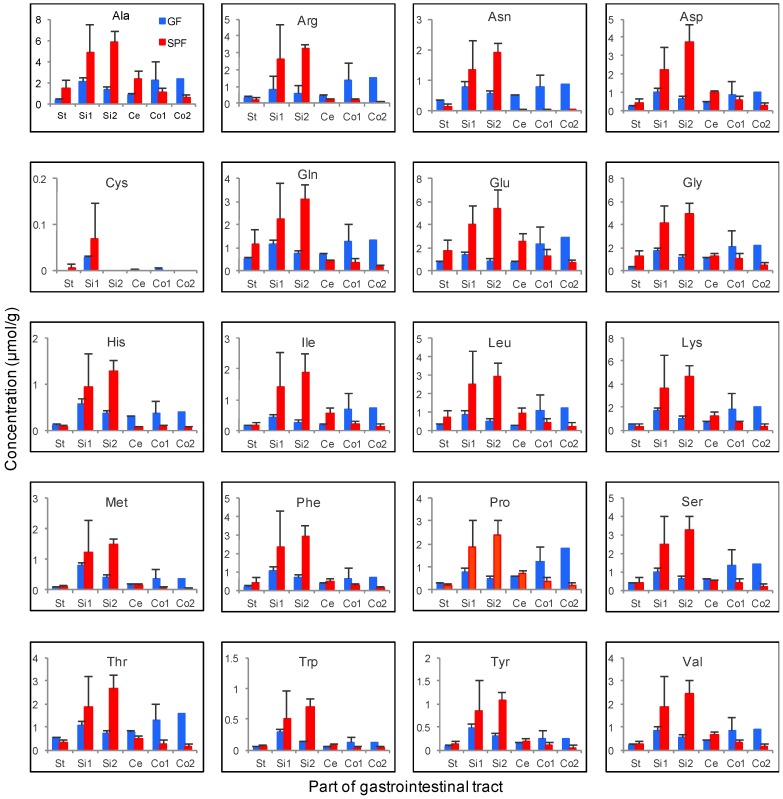
Luminal amino acid profiles of the gastrointestinal tract in SPF and GF mice. The line graphs indicate the average concentration of amino acids in the gastrointestinal contents. The red and blue lines indicate the SPF and GF mice, respectively. The error bars indicate the standard error (*n* = 3). St: Stomach, Si1: Upper small intestine, Si2: Lower small intestine, Ce: Cecum, Co1: Upper colon, Co2: Lower colon.

Our CE-TOFMS- and LC-MS/MS-based metabolome approach detected 19 sugars and 20 amino acids (Figure 5 and Figure 6). The sugar profiles were clustered into six groups based on K-means analysis (Figure 5). These six categories were different in each metabolic pattern from the stomach to the lower colon. Panose was clustered as category A. This category includes metabolites observed at higher concentrations in the stomach of SPF mice than in that of GF mice. Glucose, fructose, mannose, and ribose were included in category B; these were observed in higher concentrations in the upper small intestine of SPF mice than in that of GF mice. Mannitol, arabitol, sorbitol, raffinose, and trehalose were categorized as category C. Especially, the concentrations of mannitol, sorbitol, and raffinose were higher in the upper colon of GF mice than in the other gastrointestinal parts of GF mice. Arabitol and trehalose were present in higher concentrations in the stomach and upper small intestine of SPF mice than in those of GF mice. In addition, category D includes inositol, maltotriose, sucrose, maltose, and xylitol, which were present in higher concentrations in the stomach of SPF mice than in that of GF mice. Category E and F included two metabolites, galactose and rhamnose, and arabinose and xylose, respectively. These were present at high concentrations in the upper and lower colon of SPF mice.

Amino acid profiles from each part of the gastrointestinal tract in SPF and GF mice were drastically different (Figure 6). In SPF mice, the concentrations of all the amino acids were higher in the upper gastrointestinal tract than in the lower intestine. On the other hand, in GF mice, the concentrations of these were higher in the lower intestine than in the upper gastrointestinal tract. From these findings, the amino acids are considered to be intermediate metabolites in protein digestion and absorption by the host, in microbial digestion, and the metabolization of amino acids. 

## 4. Discussion

The types and concentrations of metabolites detected in this study varied amongst SPF and GF mice, and the average number of detected metabolites in the colon were significantly different between them (Supplementary Figure S2B). Higher numbers of microbes mainly colonized in the cecum and colon. Consistently, significantly higher amounts of unique metabolites, such as propionate and butanoate which are short-chain fatty acids (SCFAs), were detected in the SPF cecum and colon, suggesting that gut microbiota contributes to the production of cecal and colonic metabolites. It has been reported that gut microbiota–derived propionate and butanoate contribute to the progression of regulatory T cell differentiation in the murine colon, which suppresses colonic inflammation [38,42,43]. In addition, we found that significantly higher amounts of polyamines, such as putrescine, spermidine, spermine, and cadaverine, were detected in the gastrointestinal tract of SPF mice than in GF mice (Supplementary Table S1). Polyamines are well known as longevity-inducing compounds and are produced by gut microbiota [44], indicating that these compounds may contribute to health promotion in SPF mice. Furthermore, higher amounts of dicarboxylic acids, such as succinate, glutarate, adipate, pimelate, azelate, and sebacate, were detected in the gastrointestinal tract of SPF mice than in GF mice (Supplementary Table S1). It has been reported that gut microbiota-derived succinate contributes to the maturation of gut microbiota followed by protection of enteropathogenic infection [45]. Pimelate is known as an intermediate compound for biotin production in the gut [46]. Azelate is reported as whole grain-derived molecule, but it was detected only in the GF stomach and SPF gastrointestine, suggesting that azelate is not only included in whole grain, but also produced by the gut microbiota. It has been also reported that azelate could improve glucose tolerance in high fat diet-induced type 2 diabetes in C57BL/6J mice [47].

Carbohydrate-digesting enzymes, such as amylase, saccharase, and maltase, are mainly secreted in the small intestine [48]. To further understand the carbohydrate profiles throughout the gastrointestinal tract, we classified the carbohydrates detected in this study into six groups by K-means clustering (Figure 5). The metabolites identified in the stomach at the highest concentration levels in SPF mice were categorized as group A and D. Maltotriose, which is categorized in group D, is metabolized from amylose and starch [49]. Sucrose, which is also categorized in group D, is digested into glucose and fructose in the small intestine [50]. We predict that these metabolites in group D were further digested in the upper gastrointestinal tract by the host or microbiota and are absorbed by the host. As such, they are not identified in the small intestine, cecum, and colon. Group B consists of four monosaccharides. The reason why high levels of these were detected in the upper small intestine of SPF mice, but not GF mice, is likely because gut microbiota enhances digestion of dietary food, and these monosaccharides then flow into the small intestine. Group C includes carbohydrates detected at higher concentrations in the lower intestine of GF mice than in that of SPF mice. The concentrations of raffinose, sorbitol, and mannitol were higher in the lower part of the colon of GF mice than in that of SPF mice. It is estimated that these metabolites are accumulated in the upper colon because there is no bacteria in this region, and they have only a minor digestive function in the host. Groups E and F contain four carbohydrates, arabinose, xylose, galactose, and rhamnose, which are accumulated in the lower intestinal tract in SPF mice, but produced in small quantities in GF mice. The concentrations of arabinose, xylose and galactose gradually increased from the upper gastrointestinal tract to the lower gastrointestinal tract in SPF mice, suggesting that these were mainly produced from dietary food digestion by microbial carbohydrate enzymes, which may be difficult for them to be absorbed in the small intestine in mice. 

Protein-digesting enzymes such as pepsin, trypsin, chymotrypsin, and elastase are secreted in the stomach and small intestine. Comparing the gastrointestinal luminal concentrations of 20 amino acids detected in this study, we found that most of the amino acids profiles were similar (Figure 6). A relatively higher concentration of these amino acids were detected in the upper gastrointestinal tract in SPF mice, but the opposite was observed in GF mice. Based on these findings, we hypothesized that microbes contribute to protein digestion through their protein-digesting enzymes and/or enhance of protein digestion and enzyme secretion from the host. 

In this study, we revealed the differences between the gastrointestinal luminal metabolome profiles of GF and SPF mice, and highlighted the metabolic features of each part of the gastrointestinal tract. The kinds of metabolites were almost the same and characteristic metabolisms were similar between SPF and GF mice, but the metabolite concentrations and profiles throughout the gastrointestinal tract were different. This could be attributed to not just the differences in the functions of each part of the intestine, but also the colonization of microbiota. Particularly, carbohydrate, amino acid, and SCFA profiles of the SPF and GF mice were drastically different. Although correlation analysis between gastrointestinal luminal microbes and metabolites detected some specific microbe-metabolite interaction, further investigation is required for the complete understanding of the contribution of gut microbiota to the gastrointestinal luminal metabolome profiles. Our findings are considered to play an important role in understanding the relationship between commensal microbiota and gastrointestinal luminal metabolites. 

## Supplementary Materials

The following are available online at http://www.mdpi.com/2076-2607/6/4/101/s1, Figure S1: Overview of the sampling points, Figure S2: The number of detected luminal metabolites in each part of the gastrointestinal tract of SPF and GF mice, Figure S3: The composition of gastrointestinal luminal microbiota in SPF mice, Table S1: Different concentration of gastrointestinal luminal metabolites between SPF and GF mice, Table S2: The characteristic gastrointestinal luminal metabolites on each part of intestinal tract.
