# Supplementary material for: A Metabolomic-Based Evaluation of the Role of Commensal Microbiota throughout the Gastrointestinal Tract in Mice
Source: Microorganisms. 2018 Sep 29;6(4):101. doi: 10.3390/microorganisms6040101 (PMC6313407; doi:10.3390/microorganisms6040101)
Supplement: Supplementary file 1 [file microorganisms-06-00101-s001.zip › Supplementary Figures_revised.pdf]

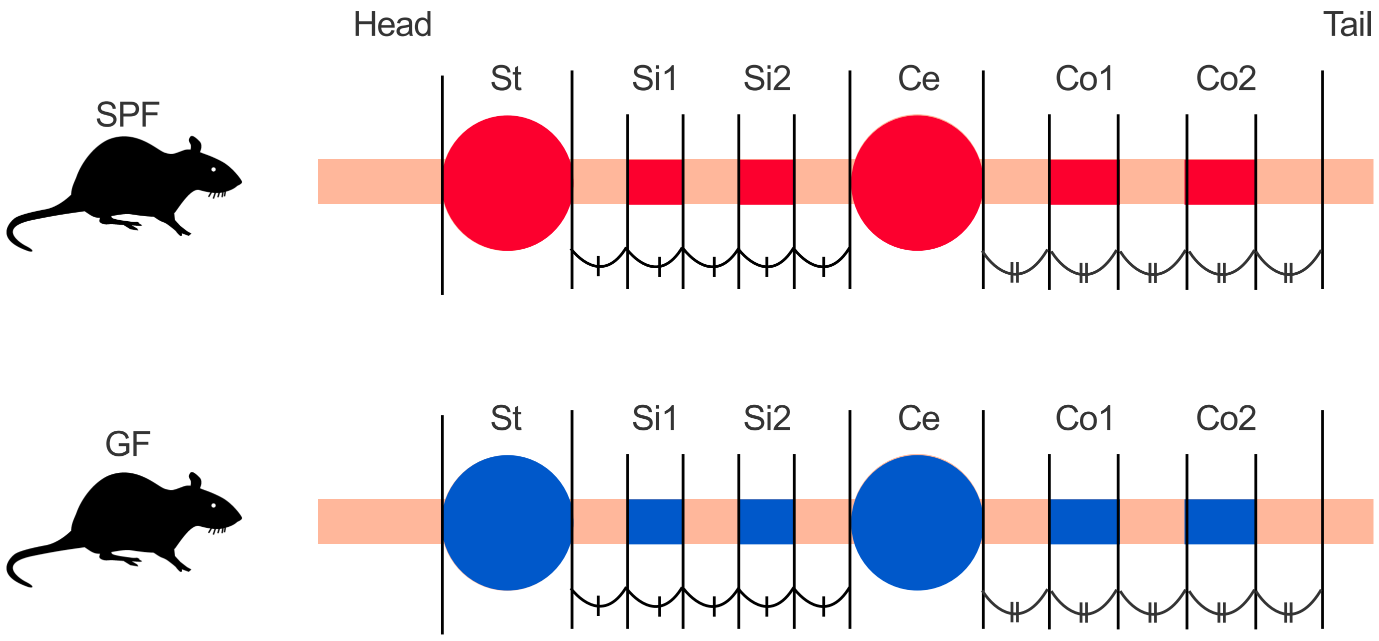

Supplementary Figure 1: Overview of the sampling points
The gastrointestinal tract was separated into the following parts and luminal contents were obtained from the colored sections (n=3). The red and blue indicate SPF and GF mice, respectively. St: stomach, Si1: upper small intestine, Si2: lower small intestine, Ce: cecum, Co1: upper colon, Co2: lower colon.

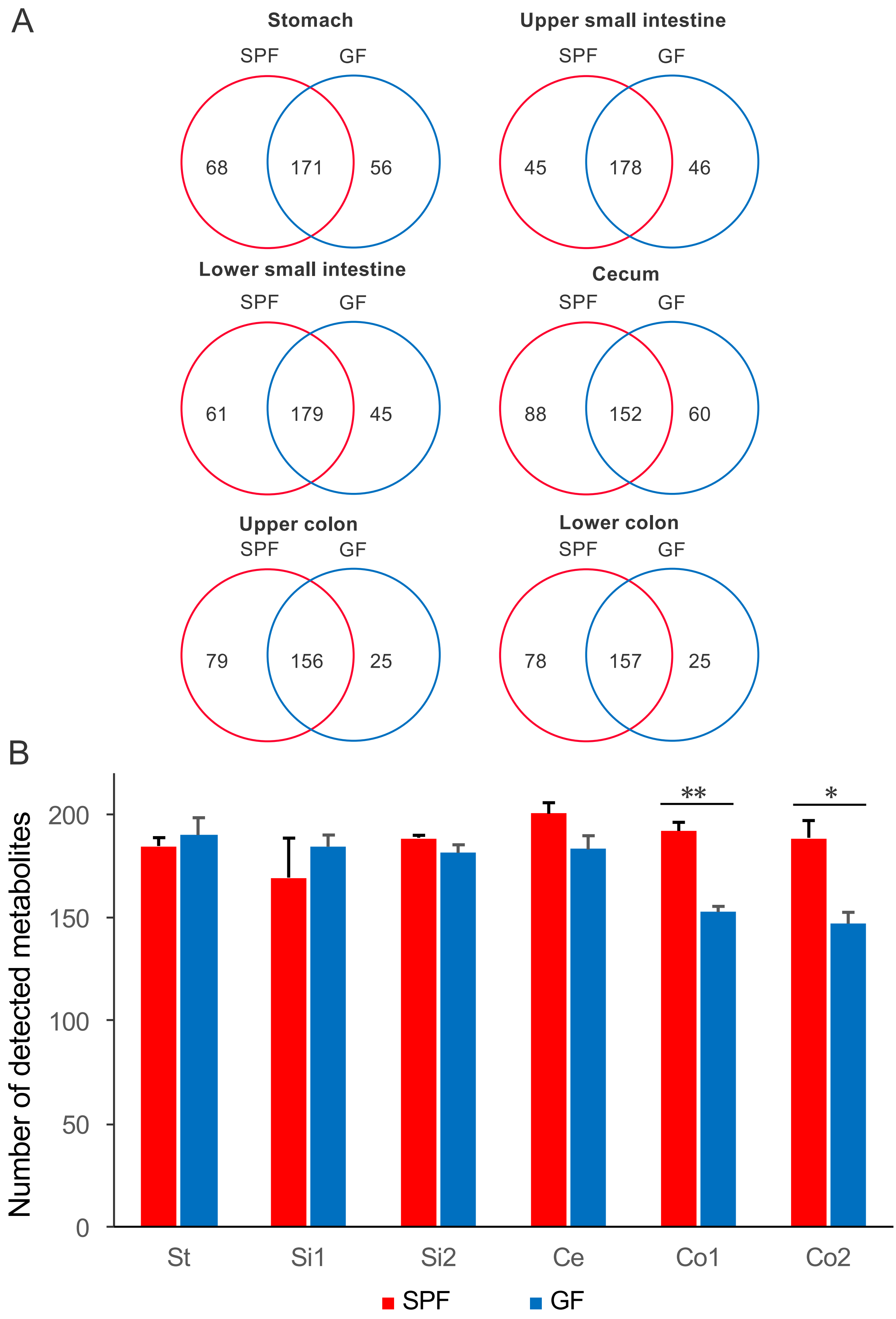

Supplementary Figure 2: The number of detected luminal metabolites in each part of the gastrointestinal tract of SPF and GF mice
A: Venn diagrams showed a total number of detected gastrointestinal luminal metabolite in SPF and GF mice. B: The average number of detected metabolites in each part of gastrointestinal tract. *P* values between SPF and GF mice were determined using Weltch's t test. **P*< 0.05; ***P*< 0.01. Error bars indicate standard error (n=3).

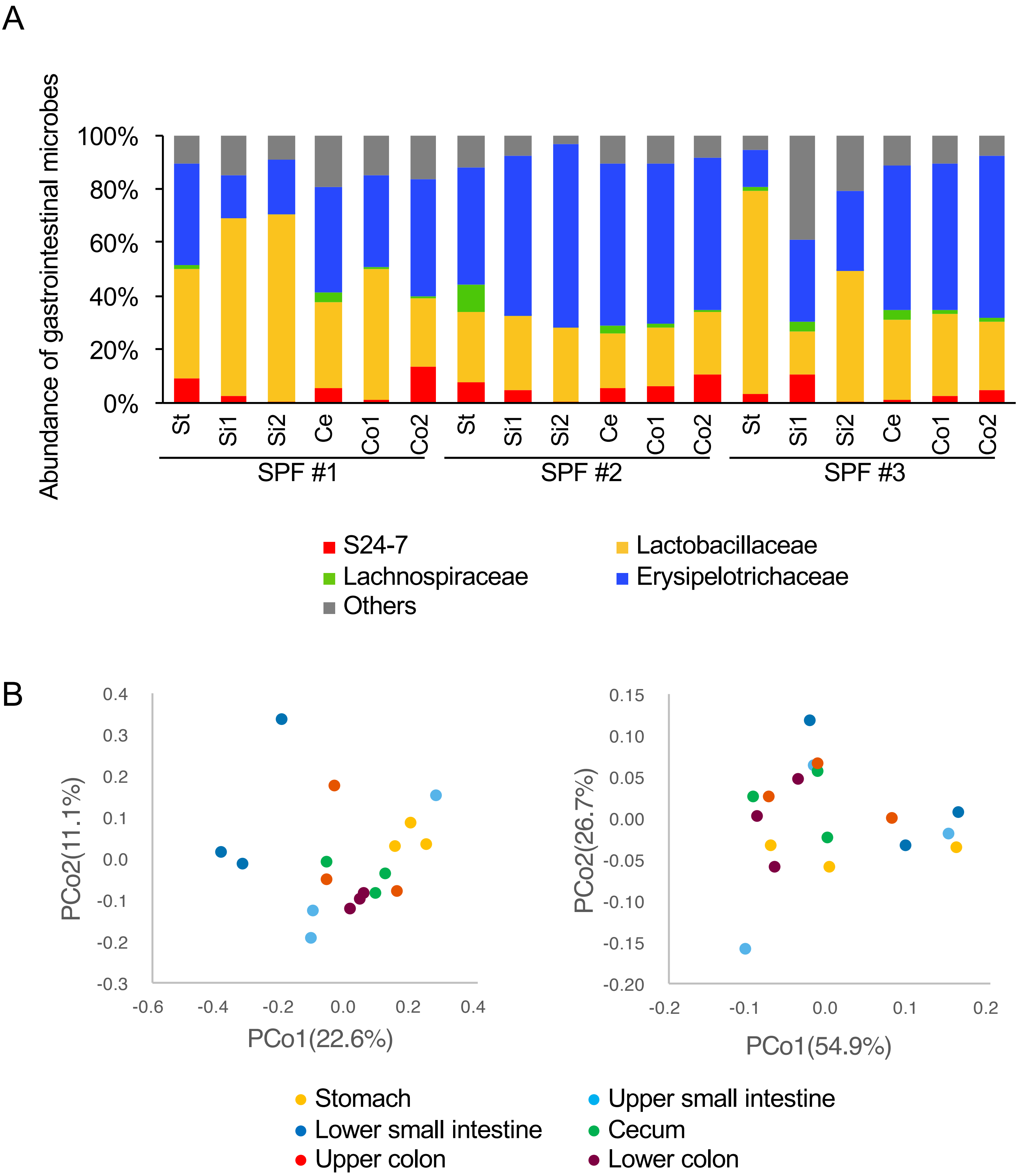

Supplementary Figure 3: The composition of gastrointestinal luminal microbiota in SPF mice

A: Vertical bars indicate relative abundances of gastrointestinal microbiota colored by microbial family. The mouse number was labeled at the bottom (n=3). St: stomach, Si1: upper small intestine, Si2: lower small intestine, Ce: cecum, Co1: upper colon, Co2: lower colon. B: PCoA plots with unweighted (left) and weighted (right) Unifrac distances.
